# The majority of patients report satisfaction more than 24 years after temporomandibular joint discectomy

**DOI:** 10.1007/s10006-024-01280-9

**Published:** 2024-07-10

**Authors:** Esmeralda Bäckström, Anders Wänman, Mats Sjöström

**Affiliations:** 1 Department of Odontology, Umeå University, Umeå, Sweden; 2 Department of Odontology, Clinical Oral Physiology, Umeå University, Umeå, Sweden; 3 Department of Odontology, Oral and Maxillofacial surgery, Umeå University, Umeå, Sweden

**Keywords:** Temporomandibular disorder, Temporomandibular joint, Discectomy, Long-term follow-up, Surgery

## Abstract

**Purpose:**

To retrospectively evaluate long-term outcomes after temporomandibular joint (TMJ) discectomy.

**Methods:**

Included patients (*n* = 64) had undergone discectomy during 1989-1998 at Umeå University Hospital. A questionnaire was used to evaluate pre- and postoperative symptoms, postoperative complications, general pain, and subjective opinion about the outcome of the surgery.

**Results:**

The results are based on responses from 47 patients (40 women/7 men), including 36 (30 women/6 men) who completed the questionnaire and 11 (10 women/1 man) who were contacted by telephone and answered selected questions. Seventeen patients were excluded because of death, a move abroad, declining to participate, or no available patient information. Among the respondents, 41 (87%) were satisfied with the results, five (11%) were unsatisfied, and one (2%) patient did not answer the question. The results showed a significant long-term improvement in locking, clicking/crepitation, and pain when chewing or opening the jaw (*p* = 0.001). The prevalence of headaches had decreased significantly at follow-up (*p* = 0.001). Reported impaired jaw-opening capacity showed no significant improvement (*p* = 0.08). Of the 47 respondents, 19 (40%) had asked for additional treatment after the discectomy, and six of the 19 patients (13%) had undergone more surgery of the joint.

**Conclusion:**

The results of this retrospective long-term follow-up study indicate that TMJ discectomy has a high success rate, as most patients were satisfied with the postoperative results. Discectomy is thus an effective surgical intervention for patients with disabling TMJ pain and dysfunction when conservative interventions have been unsuccessful.

**Supplementary Information:**

The online version contains supplementary material available at 10.1007/s10006-024-01280-9.

## Introduction

Temporomandibular disorder (TMD) is an umbrella term for diseases that cause pain or/and dysfunction in the temporomandibular joint (TMJ) or masticatory muscles [1]. The etiology of TMD is complex, not fully understood, and best comprehended in a biopsychosocial context. Biomechanical load and related microtrauma to the TMJ tissues may be a contributing factor for development of TMJ related disorders [2]. The biomechanical load may arise from primary problems in the jaws, a displaced TMJ disc, changed occlusion, or parafunctions such as grinding or clenching teeth [3]. Trauma to the TMJ without fracture can initiate TMD and has been verified as a causative factor in one-third of TMJ surgeries [4].

TMD underlies a large portion of nondental pain in the orofacial area [5, 6]. The estimated prevalence of significant TMD is 10%–15% in the adult population [1, 7]. Women are diagnosed with TMD 1.5–2 times more often than men and report more severe, frequent pain with a longer duration and distribution. The first symptoms of TMD often start at puberty [1, 8], and prevalence peaks among working-age adults [9] before beginning a decline at retirement age [8].

The most common non-invasive treatments for TMD are counseling, physiotherapy, occlusal splints, and over-the-counter medications [3, 10]. Additional treatment options include intramuscular and intra-articular injections with corticosteroids or botulinum toxin [11, 12]. If pain or dysfunction persists despite conservative treatment and significantly affects daily life, surgical procedures should be considered [3]. Between 1 and 5% of patients treated with reversible therapy are expected to need some kind of surgical intervention [12–14].

Surgical procedures vary depending on the diagnosis and comorbidities that may be related to the disability [3, 15]. For symptoms related to the TMJ, minimally invasive treatments such as arthrocentesis and arthroscopy [12] may be initial options. Discectomy, condylotomy disc reposition, or alloplastic total joint reconstruction (AJR) also are options but require open surgery [16]

Several short-term studies, with follow-up periods from 6 months to 5 years, have shown successful results of discectomy [17–19] with success rates of ~ 85% [17, 20]. In follow-up examinations, pain (muscles and jaw), chewing ability, movement of the mandibula, and horizontal maximal opening of the mouth have been evaluated in comparison with presurgical symptoms [17–21]. About 5% of patients require a second surgery to achieve the desired results [20]. Few studies have addressed outcomes at 10–30 years after surgery [22–25], however, there is a need for greater understanding of long-term outcomes after TMJ discectomies.

The aim of the current investigation was to evaluate the long-term postoperative experience and perspective after TMJ discectomy performed during 1989–1998 (i.e., 24–33 years of follow-up), with a focus on pre- and postoperative symptoms, postoperative complications, general pain, and subjective experience regarding the surgical outcome.

## Methods

### Patient cohort

**Fig. 1:**
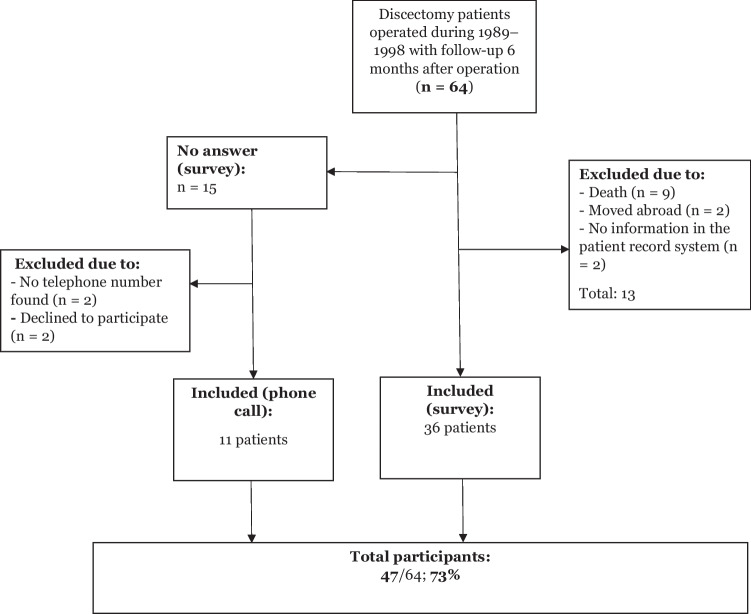
Flow chart showing inclusion and exclusion of participants in the study, resulting in a total of 47 participants

The included patient cohort has been described previously in a 6-month follow-up analysis of short-term results after discectomy in 64 patients [21]. In the current study, 13 of this original cohort of patients were excluded for the following reasons: death (n = 9), missing data in the patient record system (n = 2) and moved abroad (n = 2). The questionnaire was sent to the remaining patients (n = 51). Those who did not return the questionnaire were called by phone to answer a portion of the questions (n = 15). After agreement to participate, a total of 47 patients (mean age 61.8 [45–80] years) were included in this study, with 36 who completed the questionnaire (30 women and 6 men) and 11 who answered some questions by phone (10 women and 1 man) (Fig. 1). Pre-operative diagnosis was disc displacement with reduction in 13 patients (13/47 patients, 28%), disc displacement without reduction in 32 patients (32/47 patients, 68%) and for 2 patients no diagnosis was established (2/47 patients, 4%). Table 1 describes the internal derangements according to Wilke´s classification [26]. The classifications were evaluated individually by 2 authors (E.B.) and (M.S.). In one case there was disagreement, and consensus was reached after discussion.

**Table 1:** Wilkes classification (26) of internal derangement for the total patient cohort of 47 patients

Wilkes classification:	Number of patients n (%)
I: Early	0
II: Early/intermediate	0
III: Intermediate	19 (40%)
IV: Intermediate/late	14 (30%)
V: Late gross	14 (30%)
Total number of patients	47 (100%)

### Questionnaire

The questionnaire covered pre- and postoperative symptoms, including locking, pain from the jaw when chewing/opening the mouth, clicking/crepitation sounds from the joints, limited mouth opening, and headache from the temple areas. Other questions covered whether further treatment was needed and if the patients were satisfied with the discectomy (Supplement).

### Statistical analysis

The data were incorporated into an Excel file and transferred to the statistical program IBM SPSS Statistics (version 28.0, IBM Corp, Armonk, NY, USA). Some answers were excluded for being among multiple marked answers or because of uncertainty about which answer had been chosen. Descriptive data were analyzed using SPSS. Skewness was used to decide if the data were normally or not normally distributed. Pre- and postoperative symptoms were not normally distributed, and the samples were dependent on each other, so Wilcoxon’s signed-rank test was used. Characteristic pain intensity (CPI) was calculated as the mean score for pain right now, in general, and at its worst based on 11-point numerical pain scale where 0 represent no pain and 10 worst pain imaginable [27]. CPI scores were then classified as 0 (CPI = 0), 1 (CPI > 0 to CPI < 5), 2 (CPI > 5). To investigate differences in CPI by gender, subjective experience of the surgery, and number of postoperative symptoms, we used the non-parametric Mann–Whitney U test, as the data were not normally distributed. To calculate the association of the patients’ subjective view of discectomy and further postoperative treatment, Fisher’s exact tests were used for these categorial data. At least one of the cells was counted as less than five in all calculations. The Fisher’s exact test also was applied for comparison of the group completing the questionnaire and patients responding to questions by phone. For all analyses, significance was set at *p* < 0.05.

### Ethics

The study included gathering personal data from the patient record, and applications were sent to the ethical committee for institution of Odontology at Umeå University, the County Council of Västerbotten, and the Swedish ethical review authority (EPM). The County Council of Västerbotten approved collection of data from the participants’ electronic dental and medical records (EPM: 2022–05-09 2022–02618-01–264676). All patients received, read and signed a written consent prior to participation in the study. The consent also informed about risks/benefits of the study and the patients’ rights to stop participation in the study at any time.

## Results

Most patients (87%) were satisfied with the results of the TMJ surgery, 11% were unsatisfied, and one patient did not answer the question. Of the 36 patients who answered the questionnaire, 16 had surgery on the right side and 11 patients on the left side. Eight patients had surgery on both joints in a period of 6 months. One patient did not answer the question. Mean time for the non-surgical treatment period, prior to the discectomy, was four years and four months (range 10 months – 13 years and 11 months).

Analysis of the pre- and postoperative symptoms indicated significant post-surgery improvement in locking, pain during chewing and opening the mouth, clicking/crepitation, and headaches in the temple areas. Impaired jaw opening did not change after surgery (Table 2).

**Table 2:** Pre- and postoperative symptoms^*^

Symptoms	Preoperative n (%)	Postoperative n (%)	P^**^
Locking	29 (80.6)	2 (5.6)	0.001
Pain from jaw when chewing/opening of the mouth	31 (86.1)	15 (41.7)	0.001
Limited mouth opening	30 (83.3)	24 (66.7)	0.083
Clicking/crepitation sounds	21 (58.3)	8 (22.2)	0.001
Headache in temple areas	17 (47)	5 (13.9)	0.001
Total	36	36	

^*^Patients could register more than one symptom

^**^Wilcoxon signed-rank test

Of 47 patients, 19 reported that they had needed additional treatment after the TMJ surgery. Pain during chewing and/or opening the jaw was the most common symptom related to rescue treatment. Limited mouth opening and TMJ sounds were the next most common symptoms (60%). Headaches in the temple areas were present in 53% and locking in 33% of the patients (Table 3). In eight cases (22%), locking affected the joint where the discectomy had been performed. In one case, it affected the contralateral joint (3%), and seven patients had been seeking care for symptoms involving both joints (19%).

**Table 3:** Symptoms among the 19 patients who sought care after undergoing discectomy

Symptoms	Number of patients n (%)
Locking	5 (33.3)
Pain when chewing and/or opening the mouth	11 (73.3)
Limited mouth opening	9 (60.0)
Clicking/crepitation	9 (60.0)
Headache in temples	8 (53.3)
Other symptoms	5 (33.3)

Six patients had to undergo re-surgery because some parts of the disk had been retained or for signs of fibrous ankylosis. All six patients still used occlusal appliances after the first surgery. Half of these patients were unsatisfied with the result (Fisher’s exact test, *p* < 0.05). Patients who had needed additional care after surgery were as satisfied as patients who had not (Fisher’s exact, *p* = 0.14). Answers did not differ between those completing the questionnaires and those answering questions by phone. Among the 19 patients that needed additional treatment, the thirteen that did not undergo re-operation, were all treated with occlusal appliances (100%, 13/13) prior to the discectomy in contrast to four after the discectomy (30%, 4/13). Approximately 50% (7/13) of the 13 patients had physical therapy prior to as well as after the discectomy. Prescription of NSAID´s were identified from seven of the patients that had additional treatment and all seven (7/7) had NSAID´s prior to the discectomy compared to two patients after the discectomy (2/7) and one patent had intra-articular injection with steroids after the discectomy.

CPI was categorized into three groups to provide a comprehensive overview of the patients' overall pain. CPI = 0 was registered for 10 patients (27.8%), CPI > 0 to < 5 was registered for 15 patients (41.7%), CPI > 5 was registered for 10 patients and 1 patient had a missing value. CPI scores did not differ by gender, subjective experience of discectomy, or postoperative symptoms after the operation. However, patients who required further care postoperatively had higher CPI scores than those who did not require further care (Mann–Whitney U test, *p* < 0.05).

## Discussion

The major finding of our study is that the vast majority of patients who had undergone TMJ discectomy were satisfied with the long-term results and that their symptoms had improved after surgery. From a long-term perspective, discectomy thus can significantly improve jaw function, leading to reduced TMJ pain, locking, and clicking. This outcome corresponds with outcomes in the 6-month follow-up in this patient cohort [21]. We did not distinguish crepitation from clicking in our long-term follow-up, but there was a significant postoperative reduction in TMJ sounds. Previous studies have shown a significant reduction in clicking but not crepitation, which is mentioned as a commonly occurring symptom in other studies. Crepitation has no clear correlation with pain but is believed to be part of the remodeling process of the joint [13, 20, 28]. Headaches also decreased significantly among our patients after discectomy. One interesting observation is the long pre-operative conservative treatment period prior to the discectomy. In the current treatment regime in our team, the patient is evaluated after 6 months of conservative, for decision of eventually surgical interventions. The length of time for the pre-operative treatment may thus affect the outcome of the surgical treatment.

In the 6-month analysis of this cohort [21], mouth opening had improved significantly from a mean 36.7 mm to 41.8 mm. Half of the patients who had a jaw opening capacity of 41–60 mm before surgery had a reduced jaw opening range after surgery. This status at 6 months may underlie our current results indicating that limited opening did not improve significantly after discectomy (*p* = 0.083). These findings deviate to some extent, however, from other long-term studies that have described a jaw-opening capacity > 39 mm in most patients after surgery [22–24].

The success of discectomy depends on which parameters are chosen as outcome criteria. In our earlier retrospective 6-month follow-up study, the findings were based on patient record data from clinical examinations and followed a modified version of the American Association of Oral and Maxillofacial Surgeons criteria [21]. Our questionnaire focused on each patient’s subjective view of TMJ symptoms, and symptoms such as crepitation could have been registered differently. Most of the patients had undergone the procedure many years ago, and the precision of their memory of the experience likely varies. Recall of level of satisfaction may be accurate, but memory of specific symptoms before or after the operation may be less stable. Differences in outcomes have been noted in other studies of success rates for other treatments, with large discrepancies between the patient assessments (78%) and clinical criteria (7%) [29].

In our cohort, based on the patients’ subjective experiences after discectomy, the outcome can be viewed as satisfactory overall. Still, a considerable proportion of patients had to seek further care for persistent symptoms, most commonly pain with chewing and jaw opening. A qualitative study with interviews likely would yield further insight into their perspectives. In other long-term studies, no pain or only occasional pain was reported, with pain from the non-operated joint being more common, warranting treatment with analgesics [22, 24]. We found that pain from the operated joint was more common. Studies may differ in these outcomes because of variations in how pain was assessed, such as not presenting tenderness or stiffness as pain, which our patients may have done in responding to our questionnaire [22–24].

The patients in our study who had to seek care postoperatively had higher CPI scores for general pain at 24–33 postoperative years. These experiences may reflect other comorbidity factors such as pain in the spinal region, fibromyalgia, or depression [30–32]. It also is possible that there was some uncertainty about whether the question referred to general pain in the body or in the face region only. One register-based study showed that patients with more than one TMJ surgery had more psychiatric diagnoses and were on sick leave significantly more often than controls, indicating a more complex psychosocial comorbidity [33].

Satisfaction did not differ statistically between patients who had to seek care again and those who did not (*p* < 0.144). A possible implication is that even patients with remaining symptoms after the discectomy were still satisfied with the overall result. For the thirteen patients, which did not need re-operation, the number of further treatment decreased indicating a reduced treatment need.

Our questionnaire asked patients only to answer “yes” or “no” without any further comment or query about whether symptoms had increased or decreased postoperatively or if they were constant or occasional, as other studies have done [22–24].

Six patients (13%) had been reoperated, and they reported higher dissatisfaction with the surgical result. Approximately the same frequency of reoperations and postoperative treatments have been reported in the literature [20, 22–24, 34].

A crucial factor in these outcomes related to reoperation is postoperative rehabilitation. Rehabilitation is essential to avoid fibrous ankylosis, especially when bleeding in the joint space has occurred. Other factors also may be in play. A case–control study with prospectively collected data from Swedish national registries, analyzed the presence of mental and behavioral disorders and the probability of developing TMD. Their findings indicated an increased probability of TMD among patients with a history of certain mental and behavioral diagnoses, and a stronger association with TMD requiring surgery, specifically repeated surgery. This finding highlights the need for improved preoperative understanding of the impact of mental and behavioral conditions on TMD, as TMD and chronic pain can negatively affect mental health. Overall, it is obvious that reoperations do occur, and patients must always be informed about these risks before a discectomy [33].

One limitation of this study was the small number of reoperated patients (n = 6), which precludes drawing firm conclusions for this group. Of the overall patient cohort, 83% were women. Women tend to report severe pain more frequently than men do, which may have been a factor in our results [1, 8], although several other studies have had a similar gender distribution [16, 28, 34]. Life events that could affect discectomy outcomes also can influence long-term follow-up, including psychological factors, medication, overall pain, and comorbidity. This study lacked a control group and also does not support comparisons between discectomy and outcomes with other treatments. The authors of a systematic review comparing various surgical options concluded that despite better outcomes on some measures after discectomy, invasive surgical procedures should not be implemented as a first-line option for arthrogenous TMD management [35].

## Conclusion

The results of this retrospective long-term follow-up study of patients who had undergone discectomy indicate that the discectomies reduced symptoms indicative of TMJ disorders and that a clear majority of patients (87%) were satisfied with the results of discectomy. Future studies will assess other measures, including quality of life, function, and general pain.

## Supplementary Information

Below is the link to the electronic supplementary material.

Supplementary file1 (DOCX 267 KB)

## Data Availability

The datasets generated and analyzed during the current study are not publicly available due to the Swedish journal act but are available from the corresponding author on reasonable request.
