# Supplementary material for: The majority of patients report satisfaction more than 24 years after temporomandibular joint discectomy
Source: Oral Maxillofac Surg. 2024 Jul 10;28(4):1539–45. doi: 10.1007/s10006-024-01280-9 (PMC11480160; doi:10.1007/s10006-024-01280-9)
Supplement: Supplementary file 1 — Supplementary file1 (DOCX 267 KB) [file 10006_2024_1280_MOESM1_ESM.docx]

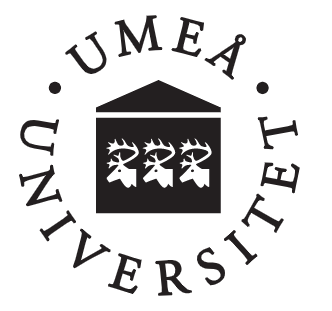
**Umeå University**
Department of Oral and Maxillofacial Surgery
Department of Odontology
901 87 Umeå

**QUESTIONNAIRE**

**LONG-TERM FOLLOW-UP FOR DISCECTOMY**

The questions in this form are about symptoms in your jaw before and after jaw joint surgery when the disk was removed, and how this has affected your jaw function and quality of life.

You have received this questionnaire because 25-35 years ago you had an operation in the jaw joint that involved removing of the disc – a so-called discectomy.

You now have the opportunity to be part of a follow-up study where we want to assess the long-term effects of the operation as well as your experiences before and after the operation. Associate Professor Mats Sjöström and Professor Anders Wänman are the supervisors who are responsible for this follow-up study.

We are aware that it has been a long time since you had surgery and we hope that you consider participating and contributing your experiences. Participation is voluntary.

If you have thoughts and concerns regarding the questions in the questionnaire, you are welcome to contact dental student Esmeralda Bäckström via telephone at 073-8761331 or e-mail at Esmeralda.backstrom@umu.se.

You will be inquired about your jaw joint symptoms before and after the operation, whether there have been any treatments in the interim from the operation until today, and how you currently assess your jaw function. Before each section, information is provided about which time interval the questions refer to.

**PART 1:** Questions in this part refer to the time BEFORE the discectomy was performed.

1. **What were your symptoms BEFORE the discectomy? Several options are possible.**

Locking of the jaw joint (difficult to fully open/close the mouth)
 Pain in the jaw joint when opening/chewing
 Limited mouth opening ability
 Snapping or cracking sounds in the jaw joint
 Headache in the temple region
 Do not remember
 Other
**If other,** give a description here: _______________________________________________________

1. **What treatment was done BEFORE the discectomy? Several options are possible.**

Informative conversation
 Movement exercises in the form of stretching, side-to-side movement, opening with resistance
 Bite guard
 Painkillers
 Cortisone injection in the jaw joint
 Grinding or building up teeth to correct the bite
 Do not remember
 Other
**If other,** give a description here: _______________________________________________________

1. **For the treatment you ticked above, how long did it last BEFORE the discectomy?**

__________years __________months

1. **How did you experience the care you received BEFORE the discectomy? Answer the following questions:**

**4a**) I felt that the staff took the time to listen to me.

Strongly agree
 Agree quite well
 Partially agree
 Disagree

**4b)** I felt that I was well informed BEFORE the discectomy and knew why I was having the surgery.

Strongly agree
 Agree quite well
 Partially agree
 Disagree

**4c)** Before the operation I felt safe with the staff and trusted their competence.

Strongly agree
 Agree quite well
 Partially agree
 Disagree

1. **Did you feel you were missing anything BEFORE the operation? What could have been improved?**

_________________________________________________________________________________________________________________________________________________________________________________________________________________________________

1. **What did you appreciate about the staff BEFORE you had surgery?**

_________________________________________________________________________________________________________________________________________________________________________________________________________________________________

**Part 2:** Questions in this part refer to the time IMMEDIATELY AFTER the discectomy was performed.

1. **What symptoms did you have IMMEDIATELY AFTER the discectomy?**

None
 Locking of the jaw joint (difficult to fully open/close the mouth)
 Pain in the jaw joint when opening/chewing
 Limited mouth opening ability
 Snapping or cracking sounds in the jaw joint
 Headache in the temple region
 Other
**If other,** give a description here: **_______________________________________________________**

1. **How did you experience the care you received IMMEDIATELY AFTER the discectomy? Answer the following questions:**

**2a)** I felt that the staff took the time to listen to me.

Strongly agree
 Agree quite well
 Partially agree
 Disagree

**2b)** I felt that the staff cared about me AFTER the operation.

Strongly agree
 Agree quite well
 Partially agree
 Disagree

**2c)** I felt that I had been given information about where to go AFTER the surgery if my symptoms returned.

Strongly agree
 Agree quite well
 Partially agree
 Disagree

1. **Did you feel you were missing anything IMMEDIATELY AFTER the operation (e.g. more painkillers, the care of the staff)? What could have been improved?**

_________________________________________________________________________________________________________________________________________________________________________________________________________________________________

1. **What did you appreciate during the period RIGHT AFTER you had surgery?**

_________________________________________________________________________________________________________________________________________________________________________________________________________________________________________________

**Part 3 -** Questions in this part refer to the INTERMEDIATE PERIOD after the operation (discectomy) until today.

1. **How would you describe your general state of health during the period from the discectomy until today?**

Very bad
 Bad
 Average
 Good
 Very good

1. **Have you had to seek help again after the operation (excluding rehabilitation) due to problems with your jaw joint?**

Yes
 No

**If Yes, answer the following questions (2a-2f), otherwise skip to question 3.**

**2a) Which problems did you seek help for?**

Locking of the jaw joint (difficult to fully open/close the mouth)
 Pain in the jaw joint when opening/chewing
 Limited mouth opening ability
 Snapping or cracking sounds in the jaw joint
 Headache in the temple region
 Do not remember
 Other
**If other**, give a description here: _______________________________________________________

**2b) Which jaw joint did the problems concern?**

The operated jaw joint
 The non-operated jaw joint
 Both the operated and non-operated jaw joints

**2c) What type of treatment did you receive after the surgery? Several options are possible.**

Informative conversation
 Movement exercises in the form of stretching, side-to-side movement, opening with resistance
 Bite guard
 Painkillers
 Cortisone injection in the jaw joint
 Grinding or building up teeth to correct the bite
 Additional jaw joint operation (s)
 Other
**If other**, give a description here: ______________________________________________________

**2d)** **When did you start this treatment?**
Give the year ____________________

**2e) How long did the treatment last? If you are currently undergoing treatment, write Pending.**____________________

**2f)** **Were the problems so extensive that you needed to operate the jaw joint again?**
 Yes
 No

**3. Have you recently had a blow or injury to your face or jaw where you have had residual pain/problems?**

Yes
 No

1. **If yes, did you have these problems/pains before the injury to your face?**

Yes
 No

**Pain and its Consequences:**

**5.** **Is your pain continuous, recurring, or occurs only now and then?** Continuous
 Recurring regularly
 A single occasion now and then

**6.** **How would you rate your pain right now in the face/mouth? Rate on a scale of 1-10, where 0 corresponds to "no pain" and 10 corresponds to "unbearable pain".**

No pain 0 1 2 3 4 5 6 7 8 9 10 unbearable pain

**7.** **In the last 6 months, how intense was the pain (on average) when it was at its worst? Rate on a scale of 1-10, where 0 corresponds to "no pain" and 10 corresponds to "unbearable pain".**

No pain 0 1 2 3 4 5 6 7 8 9 10 unbearable pain

**8.** **In the last 6 months, how intense was the pain (on average) on a scale of 0-10, where 0 corresponds to "no pain" and 10 corresponds to "unbearable pain"?**

No pain 0 1 2 3 4 5 6 7 8 9 10 unbearable pain

**9.** **How many days in the last month have you refrained from your usual activities (work, school, housework) because of pain in your face/mouth? _______Days**

**10.** **In the last 6 months, how much has pain in the face/mouth disturbed (impeded) your daily activities on a scale of 0-10, where 0 corresponds to "no disturbance" and 10 corresponds to "unable to perform any activities"?**

No disturbance 0 1 2 3 4 5 6 7 8 9 10 unable to perform
            any activities

**11. In the last 6 months, how much has the pain in the face/mouth changed your ability to participate in leisure, social and family activities? Rate on a scale of 0-10, where 0 corresponds to "no change" and 10 corresponds to "complete change".**

No 0 1 2 3 4 5 6 7 8 9 10 Complete
change            change

**12. In the last 6 months, how much has the pain in the face/mouth changed your ability to work (including housework)? Rate on a scale of 0-10, where 0 corresponds to "no change" and 10 corresponds to "complete change"”.**

No 0 1 2 3 4 5 6 7 8 9 10 Complete
change            change

**13. Which other areas do you have pain in right now?**
 Hands  Elbow  Shoulders  Neck  Upper Back  Lower Back  Chest  Stomach  Hips  Knees  Feet

**Pain drawing distribution**

Where do you experience pain? In the figures below, illustrate your pain location and spread by shading in the areas that show it best. If the pain is exact, you can draw it in with a dot. If the pain moves from one place to another, show with arrows the direction the pain moves.


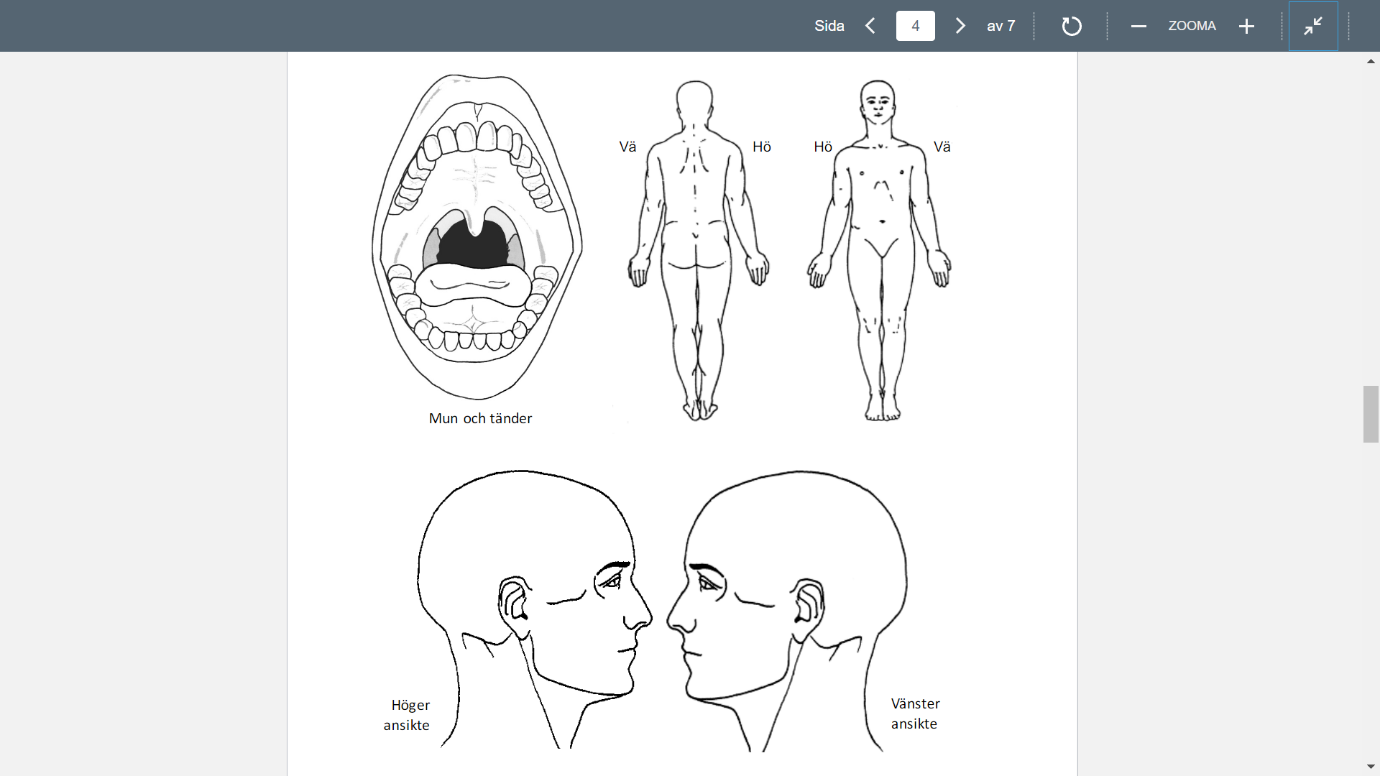


**OHIP – Oral Health Impact Profile**

Below are descriptions of 14 different situations that may be related to your teeth, your mouth, your prostheses or your jaws. How often in the last month have you experienced the following situations due to problems with your teeth, mouth or jaws? For each statement, mark the option that best corresponds to your experience. Use "does not apply" if you believe that the statement does not pertain to you.

|  |  | Does not apply | Always | Very often | Rather often | Sometimes | Rarely | Never |
| --- | --- | --- | --- | --- | --- | --- | --- | --- |
| A | Difficulty pronouncing words |  |  |  |  |  |  |  |
| B | Have had pain in the mouth |  |  |  |  |  |  |  |
| C | Have had discomfort in eating food |  |  |  |  |  |  |  |
| D | Have had unsatisfactory food |  |  |  |  |  |  |  |
| E | Feeling tense |  |  |  |  |  |  |  |
| F | Have had to cancel the meal |  |  |  |  |  |  |  |
| G | Have had a hard time relaxing |  |  |  |  |  |  |  |
| H | Been embarrassed |  |  |  |  |  |  |  |
| I | Had difficulty performing daily tasks |  |  |  |  |  |  |  |
| J | Felt that life in general has been less satisfying |  |  |  |  |  |  |  |
| K | Have been totally unable to function |  |  |  |  |  |  |  |
| L | Felt that the ability to taste has changed |  |  |  |  |  |  |  |
| M | Feeling insecure |  |  |  |  |  |  |  |

**JFLS-20 Jaw function limitation scale**

For each question below, indicate the degree of limitation in the jaws during the past month by placing a check mark in the box that best describes it. If it was impossible to carry out the activity, put a cross in the box on the far right (full limitation).

|  |  | No limitation |  |  |  |  |  |  |  |  |  | Full limitation |
| --- | --- | --- | --- | --- | --- | --- | --- | --- | --- | --- | --- | --- |
|  |  | 0 | 1 | 2 | 3 | 4 | 5 | 6 | 7 | 8 | 9 | 10 |
| 1 | Chew tough food |  |  |  |  |  |  |  |  |  |  |  |
| 2 | Chew hard bread (e.g. crackers) |  |  |  |  |  |  |  |  |  |  |  |
| 3 | Chew chicken (e.g. cooked in the oven) |  |  |  |  |  |  |  |  |  |  |  |
| 4 | Chew biscuits |  |  |  |  |  |  |  |  |  |  |  |
| 5 | Chew soft food (e.g. macaroni, cooked vegetables, fish) |  |  |  |  |  |  |  |  |  |  |  |
| 6 | Eat soft food that does not need chewing (e.g. mashed potatoes, apple custard, pudding) |  |  |  |  |  |  |  |  |  |  |  |
| 7 | Mouth opening wide enough to bite an apple |  |  |  |  |  |  |  |  |  |  |  |
| 8 | Mouth opening wide enough to bite a sandwich |  |  |  |  |  |  |  |  |  |  |  |
| 9 | Mouth opening wide enough to speak |  |  |  |  |  |  |  |  |  |  |  |
| 10 | Mouth opening wide enough to drink from a mug |  |  |  |  |  |  |  |  |  |  |  |
| 11 | Swallow |  |  |  |  |  |  |  |  |  |  |  |
| 12 | Yawn |  |  |  |  |  |  |  |  |  |  |  |
| 13 | Speak |  |  |  |  |  |  |  |  |  |  |  |
| 14 | Sing |  |  |  |  |  |  |  |  |  |  |  |
| 15 | Look happy |  |  |  |  |  |  |  |  |  |  |  |
| 16 | Look angry |  |  |  |  |  |  |  |  |  |  |  |
| 17 | Snorting |  |  |  |  |  |  |  |  |  |  |  |
| 18 | Kissing |  |  |  |  |  |  |  |  |  |  |  |
| 19 | Smiling |  |  |  |  |  |  |  |  |  |  |  |
| 20 | Laughing |  |  |  |  |  |  |  |  |  |  |  |

**General questions:**

1. **I define myself as**

Woman
 Man

1. **My age is (give number)**

_______ year

1. **Which jaw was operated on?** Right Left
   If you operated on both joints, tick
   both squares
2. **Are you satisfied with the result of the discectomy?** Yes No

1. **Do you regret having the discectomy?** Yes No

**5a. If yes, why do you regret having a discectomy??**
 ________________________________________________

1. **Would you choose to have surgery again if needed?** Yes No

**6a.** Describe the change in **activity restriction** (e.g. abstaining from social events, dinners, exercise, etc.) BEFORE the discectomy compared to AFTER treatment until today.

 Refrain from most activities
 Unchanged
 Can take part in more activities

**6b.** Describe the change in **emotional state** BEFORE the discectomy compared to AFTER treatment until today.
 Negative impact
 Unchanged
 Positive impact

**6c.** Describe the change in life quality BEFORE the discectomy compared to AFTER treatment until today.

Poorer quality of life
 Unchanged quality of life
 Increased quality of life with significant improvement that made a big difference

1. **Is there anything else you want to add regarding your jaws or the operation?**

__________________________________________________________

**If it becomes relevant, could we contact you for a supplementary interview?
If YES, write the information where we can contact you (phone or email)**

__________________________________________________________

Thank you for your participation!

If you have any questions, please contact Esmeralda Bäckström
**Tel: 073-8761331 or email:** [Esmeralda.backstrom@umu.se](mailto:Esmeralda.backstrom@umu.se)
